# Antioxidative Effects of Olive Polyphenols Compared to Vitamin E in Piglets Fed a Diet Rich in N-3 PUFA

**DOI:** 10.3390/ani9040161

**Published:** 2019-04-12

**Authors:** Jakob Leskovec, Vida Rezar, Alenka Nemec Svete, Janez Salobir, Alenka Levart

**Affiliations:** 1 Department of Animal Science, Biotechnical Faculty, University of Ljubljana, 1000 Ljubljana, Slovenia; jakob.leskovec@bf.uni-lj.si (J.L.); vida.rezar@bf.uni-lj.si (V.R.); janez.salobir@bf.uni-lj.si (J.S.); 2 Small Animal Clinic, Veterinary Faculty, University of Ljubljana, 1000 Ljubljana, Slovenia; alenka.nemecsvete@vf.uni-lj.si

**Keywords:** hydroxytyrosol, linseed oil, olive polyphenols, oxidative stress, piglets, vitamin E

## Abstract

**Simple Summary:**

Olive polyphenols exert many favorable properties, such as an antioxidative effect in humans, but are poorly studied in animals, especially in pigs. Therefore, we conducted a trial including olive leaf extract (OLE), which is rich in polyphenols, in pigs fed a linseed oil-enriched diet, which is a known cause of postprandial oxidative stress. OLE exerted some antioxidative effects in piglets fed a polyunsaturated fatty acid (PUFA)-rich diet without any negative effects, even when included in a higher concentration than that proposed for humans. Additionally, the inclusion of OLE did not compromise the zootechnical performance of the piglets as in the case of the dietary inclusion of olive leaves. Therefore, OLE could be used as an effective feed additive for beneficial effects on intestinal health and meat quality, among others.

**Abstract:**

The aim of the current study was to assess the antioxidative effects of the dietary supplementation of olive leaf extract (OLE) in different concentrations compared to those of vitamin E in piglets under conditions of dietary n-3 PUFA-induced oxidative stress. Forty-eight castrated male piglets (10.6 ± 0.99 kg) were fed the following experimental diets: Cont− (low-fat diet, no supplement), Cont+ (high linseed oil diet, no supplement), Vit-E (as Cont+, 105 IU vitamin E/day), OLE-1 (as Cont+, 3.84 mg hydroxytyrosol equivalents (HEQs)/day), OLE-2 (as Cont+, 38.4 mg HEQ/day), and OLE-3 (as Cont+, 96 mg HEQ/day). After 21 days of feeding, the experimental diets, blood and urine samples were collected to assess the extent of the oxidative stress. Results indicated that diet OLE-1 lowered the activity of gamma-glutamyl transferase, protected DNA (measured as DNA tail %) and altered urinary 8-hydroxy-2’-deoxyguanosine (8-OHdG). Dietary vitamin E lowered the levels of urinary F_2_-isoprostanes, as well as of plasma malondialdehyde and γ-tocopherol, but raised the plasmatic α-tocopherol and altered the level of urinary 8-OHdG. In conclusion, only minor positive effects of dietary OLE on the oxidative stress parameters were observed. Additionally, OLE did not show concentration dependence.

## 1. Introduction

Olive plant (*Olea europaea* L.) contains different polyphenols, of which some are suggested to have antioxidative and other beneficial properties in humans [1] and possibly in farm animals—according to scarce literature data. The effectiveness of hydroxytyrosol and its derivatives as antioxidants in humans is acknowledged by the European Food Safety Authority through a health claim, which states that 5 mg of hydroxytyrosol equivalents (EQs) ingested with olive oil can protect low-density lipoproteins (LDL) from oxidation [2]. 

To the best of our knowledge, no studies in the literature have thoroughly evaluated the potential of olive polyphenols to reduce oxidative stress in farm animals. Trials with a limited number of observed parameters were conducted to study the antioxidant effects of olive polyphenols in pigs. Results were inconclusive, since dietary olive leaves in 5–10% concentrations showed no clear effects on lipid peroxidation [3]. However, Botsoglou et al. [4] reported that the dietary supplementation of 0.5 and 1% olive leaves decreased lipid oxidation in pork, measured as malondialdehyde (MDA). 

The dietary inclusion of olive leaves as a source of olive polyphenols in pigs is questionable, since it may have negative effects on feed intake and growth performance [3,5]. Therefore, the use of polyphenol-rich olive extracts could be beneficial but has not been studied in pigs. In addition, no data on the effect of various dietary concentrations of olive polyphenols are known, and the recommendations for growing pigs are most likely different than for humans.

Thus, the objective of this study was to determine the antioxidative effects of the increasing dietary inclusion of olive leaf extract (OLE) as a source of polyphenols, and to compare them to the effects of a supranutritional vitamin E supplementation in piglets. In order to measure the possible antioxidant effects of olive polyphenols, the oxidative load was increased via a PUFA-rich diet, known to induce postprandial oxidative stress in pigs, and therefore an additional supplementation with antioxidants is beneficial [6]. Since oxidative stress induces damage to different macromolecules, which might lead to increased apoptosis and tissue damage [7], a broad set of markers of lipid oxidation, levels of antioxidants, as well as of antioxidant enzymes, liver enzymes, and DNA damage were used.

## 2. Materials and Methods 

### 2.1. Animals, Experimental Design and Dietary Treatments

Forty-eight weaned and castrated commercial male piglets (Slohibrid commercial pigs—Slovenian Landrace, Slovenian great white pig and Duroc) were used in the trial, which was divided into two repetitions with twenty-four animals. The piglets (10.6 ± 0.99 kg) were assigned to six experimental groups and fed the experimental diets once per day for 21 days. The animals were caged individually in balance cages (850 mm × 530 mm × 760 mm), which permitted the individual collection of urine samples. The temperature was kept at 28 °C in the beginning and was gradually lowered to 24 °C, while light was provided for 13 hours per day (07:00–20:00 h). The experimental design and protocol were approved by the Animal Ethics Committee of the Veterinary Administration of Slovenia (U34401-9/2016/7).

The experimental feed was formulated according to the Nutrition requirement of swine [8] and was fed at a level of 2.5 times the maintenance energy requirements [8] (Table 1) in order to ensure the animals consumed the feed completely. Pigs in the negative control group were fed a low-fat diet (0.6% of energy from n-3 PUFA; Cont−), while the other five groups were fed a high-fat diet enriched with 8.3% of linseed oil (10.4% of the provided energy from n-3 PUFA) (Table 1 and Table 2). In the high-fat diet groups, Cont+ had no supplementation, Vit-E was supplemented with 105 IU vitamin E/day (as DL-α-tocopheryl acetate), while the remaining three diets were supplemented with an OLE (OLE-1, OLE-2, OLE-3). OLE was added in three different concentrations, the lowest one following the recommendation for humans [2], which maintains that 5 mg of hydroxytyrosol EQs per day protects LDL from oxidative damage. The amount of OLE in OLE-1 was adjusted to the energy intake of pigs (3.84 mg of hydroxytyrosol EQ/day), calculated as follows: the daily dose in humans is 5 mg of hydroxytyrosol. By energy requirements: 10.5 MJ ME per day = 0.48 mg hydroxytyrosol/MJ; piglets consumed 8 MJ ME/day = 3.84 mg hydroxytyrosol EQ per day (0.34 g of OLE). The hydroxytyrosol concentration in the OLE-2 and OLE-3 diets (38.4 mg, 3.4 g OLE, and 96 mg of hydroxytyrosol EQ, 8.5 g OLE, per day, respectively) was 10 and 25 times higher than in OLE-1 (Table 2). To prevent oxidative deterioration, linseed oil and OLE were added and mixed into the diets directly before feeding. The live weight of the piglets was monitored weekly and the amount of feed was recalculated and adjusted accordingly. Feed conversion was calculated as total feed intake/total weight gain.

### 2.2. Preparation and Standardization of Olive Leaf Extract

Olive leaves collected from an organic olive grove were dried at 45 °C, milled (hammer mill, particle size <2 mm), immersed in 70% ethanol (250 g leaves/L) and shaken on an orbital stirrer for four days. After extraction, the mass was filtered through a gauze and evaporated at 40 °C to 15% of the initial weight. Then, 20% propylene glycol:water (1:1) was added in order to dissolve the residuals. The final extract contained 1.70% oleuropein, 0.64% hydroxytyrosol and 5.85% total polyphenols (as oleuropein equivalents), determined according to the official method of the International Olive Oil Council (COI/T.20/Doc no. 29). Hydroxytyrosol equivalents (EQs) were calculated as the sum of (free) hydroxytyrosol and hydroxytyrosol contained in oleuropein, using the stoichiometric ratio between the two.

### 2.3. Sample Collection

At the end of the trial, blood from the jugular vein was collected into Vacuette tubes (Greiner Bio-One, Cassina de Pecchi, Italy). The plasma was collected in K3-EDTA tubes and centrifuged (1800 g for 15 min at 4 °C) to conduct the analysis of MDA, α- and γ-tocopherols. Whole blood was used to conduct the analyses of hemoglobin and comet assay. The serum for the analyses of the antioxidant capacities of water (ACW)- and lipid (ACL)-soluble antioxidants, activities of aspartate aminotransferase (AST), alanine aminotransferase (ALT) and gamma-glutamyl transferase (GGT), as well as oxidized LDL (oxLDL), was taken into collection tubes with a clot activator, left for 2 h at room temperature and centrifuged at 2500 g for 10 min at 20 °C. Lithium heparin tubes were used to collect whole blood for the analyses of the activities of superoxide dismutase (SOD) and glutathione peroxidase (GPx). 

Urine samples were collected from days 17 to 19 (48 h) of the experimental period. Urine samples were collected in polypropylene containers on a daily basis and stored at 4 °C. Total collected urine samples (48 h) were weighted, filtered through a filter paper, purged with a nitrogen stream and stored in polypropylene micro-centrifuge tubes. The addition of 0.005% of butylated hydroxytoluene and 0.05% sodium azide for the analyses of F_2_-isoprostanes and 8-OHdG, respectively, was performed prior to storage at −80 °C.

### 2.4. Chemical Analyses

Feed samples were analyzed weekly for dry matter, crude protein, crude fat, crude ash, acid and neutral detergent fiber (ADF and NDF), Ca and P using the methods of Naumann and Bassler [9]. Plasma and urinary MDA levels, together with plasma α- and γ-tocopherol levels, were determined following the methods of Voljč et al., [10]. Photochemiluminiscence was used to determine ACW and ACL following the protocol provided by the manufacturer (Photochem, Analytik Jena, Jena, Germany). Liver enzymes in serum (AST, ALT, and GGT) and the activities of SOD and GPx in whole blood were determined with an automated clinical chemistry analyzer (RX-Daytona, Randox, Crumlin, UK). The activities of SOD and GPx were expressed as units per gram of hemoglobin (U/g HGB) determined by the hematological analyzer ADVIA 120 (Siemens, Munich, Germany). Oxidized LDL were determined with an ELISA kit (CSB-E 06527p, Cusabio Biotech, Wuhan, China), while the comet assay (DNA tail % and olive tail moment, OTM; product of the tail length and the fraction of total DNA in the tail) was performed following the method by Frankič et al., [6] except for the image analysis, which was performed with different software (Komet, version 7.0.1 08, Andor Technology Ltd., Belfast, UK) and DNA dye (GelRed, Biotium, CA, USA). F_2_-isoprostanes (iPF2α-III) were measured by a commercial ELISA kit (no. 516351, Cayman Chemical, Ann Arbor, MI, USA), and 8-OHdG by the ELISA kit ADI-EKS-350 (Enzo Life Science, Farmingdale, NY, USA). The fatty acid profile was measured as described by Frankič and Salobir [11].

### 2.5. Statistical Analysis

For the analyses of variance, the mixed procedure from SAS software was used (ver. 9.4, Sas Inc., Cary, NC, USA). In the statistical model, the experimental groups, the replicates and the interaction between the two were used as fixed effects, while an individual animal was considered as an experimental unit. The repeated statement was used to include the analytical variability within an experimental unit. For the analyses of the results of the comet assay, the random statement was used to include the measured values of the cells within an experimental unit. The heterogeneous variance was presumed in the observed parameters and differences between the groups were tested with the Tukey–Kramer multiple-comparison test. The least square means and the SEMs as a dispersion parameter are shown in the results section. Statistical significance was accepted at *p* < 0.05 (*p* value for the Tukey–Kramer multiple comparison test), and the actual P-value is given for tendencies. 

## 3. Results

### 3.1. Growth Performance

During the trial, the animals reached the expected growth. No differences (*p* > 0.05) in the final body weight or body weight gain were obtained. Animals fed Cont− had higher (*p* < 0.05) feed intake and poorer conversion ratio in comparison to the groups fed linseed oil-supplemented feeds (Table 3).

### 3.2. Extent of Lipid Oxidation

Lower urinary excretion of F_2_-isoprostanes in piglets fed diet Vit-E than in those fed Cont− (*p* < 0.05) was detected, while no differences were obtained between the other groups. Regarding plasma MDA levels, they were higher in piglets fed Cont+ (*p* < 0.05) than in piglets fed Cont−. The latter, in turn, had lower (*p* < 0.05) plasma MDA levels than OLE-1, OLE-2, and OLE-3. A statistical tendency (*p* = 0.104) was obtained between the plasma MDA levels of animals fed Vit-E and those of animals fed Cont+, with the first being lower. Piglets fed Vit-E also had lower plasma MDA levels than those fed OLE-1 (*p* < 0.05). The supplementation with OLE did not affect plasma MDA levels in comparison to Cont+. In relation to the serum oxLDL concentration and urinary MDA excretion levels, no differences were observed between groups, although statistical tendencies were obtained between the urinary MDA excretion levels of piglets fed Cont− and those fed Cont+ (*p* = 0.115) and OLE-3 (*p* = 0.074) (Table 4), the first being lower.

### 3.3. Level of Antioxidants and Antioxidant Enzymes

There were no differences in plasma α-tocopherol between piglets fed Cont−, Cont+ and OLE-supplemented feeds, while Vit-E increased the level of plasma α-tocopherol (*p* < 0.05) in comparison to these groups. Piglets fed Cont− had a lower level of plasma γ-tocopherol than those fed Cont+, while piglets fed Vit-E had a lower level of plasma γ-tocopherol than those fed Cont−, Cont+ and OLE-supplemented diets. By means of the determination of the ACW, we observed a tendency of animals fed Cont− to have a lower ACW than those fed OLE-2 (*p* = 0.078). Analyses of the GPx activity resulted in higher GPx activity in animals fed Vit-E than those fed OLE-1 (*p* = 0.096) and OLE-2 (*p* = 0.083). Serum ACL and SOD activity did not differ between the groups (Table 5).

### 3.4. Rate of DNA Damage and Activity of Liver Enzymes 

Animals fed Cont− had a lower (*p* < 0.05) tail DNA % than those fed Cont+ and Vit-E, but not than those fed the OLE-supplemented feed, regardless of the inclusion rate. Regarding the OTM, the only difference obtained was the higher value observed in piglets fed Vit-E (*p* < 0.05) compared to those fed Cont−, Cont+ and OLE-supplemented feeds. Urinary 8-OHdG excretion was higher (*p* < 0.05) in animals fed Cont− than in those fed Cont+, OLE-2 and OLE-3. Moreover, piglets fed Vit-E had a higher excretion of 8-OHdG (*p* < 0.05) than those fed Cont+, OLE-2 and OLE-3. However, the 8-OHdG excretion of pigs fed the OLE-supplemented diet did not differ from that of piglets fed Cont+. Serum GGT activity was lower in animals fed Cont− and OLE-1 than in those fed Cont+ (*p* < 0.05), while there were no effects on serum GGT activity of animals fed Vit-E, OLE-2 and OLE-3. No differences in serum AST and ALT were found between the groups (Table 6).

## 4. Discussion

Even though olive polyphenols have many beneficial effects on animal and human health [1,2,3,5], only a few trials have investigated their use as antioxidants in pigs. Moreover, the potential of extracted olive polyphenols at different dietary levels has not been studied in detail in animals. 

In the present study, oxidative stress was induced in Cont+, Vit-E and the three OLE-supplemented groups by including high linseed oil, which was exchanged for wheat starch in the diet fed to the Cont− group. Consequently, Cont− feed contained less energy than Cont+, and therefore a higher feed intake and a poorer feed conversion ratio were observed in animals fed Cont− than in the rest. Moreover, dietary supplementation with OLE did not affect the growth performance of the piglets compared to Cont+, contrarily to the results obtained in pigs fed dietary olive leaves [3,5]. The inclusion of OLE did not affect the energy content of the feed or antinutritive components, as might be the case with olive leaf supplementation. In spite of this, further studies under practical rearing conditions without feed restriction should be performed to confirm our hypothesis, since the inclusion of OLE could affect feed consumption, growth and feed conversion, as reported for some polyphenols [12].

One of the most accepted markers of in vivo oxidative stress is urinary excretion of F_2_-isoprostanes [13]. We did not detect any differences in urinary excretion of F_2_-isoprostanes between Cont− and Cont+, suggesting that oxidative stress caused by the high PUFA intake was not very pronounced. Nevertheless, dietary OLE did not have an effect on F_2_-isoprostanes, which is in agreement with studies in humans [2], although some trials showed lower excretion of isoprostanes in humans after the consumption of olive polyphenols [14]. In the present study, the only reduction of F_2_-isoprostanes was observed with the inclusion of vitamin E, which is in line with some trials in humans [15]. F_2_-isoprostanes could be a biased marker of oxidative stress, since the linseed oil-supplemented feeds had different n-6:n-3 PUFA ratios (0.54:1) in comparison to Cont− (5.3:1). The differences in the dietary linoleic fatty acid, which is elongated to arachidonic fatty acid, the precursor of F_2_-isoprostanes, were reflected in the fatty acid composition of the erythrocytes. Animals in group Cont− had a higher proportion of arachidonic acid in the erythrocytes (Cont− = 4.93 wt %; n = 4) than the linseed oil-supplemented groups (Cont+ = 2.75 wt %; n = 4), and therefore higher F_2_-isoprostane levels in Cont− were expected. Therefore, it is not clear if the differences were due to the difference in the level of arachidonic acid in the body or to the lower oxidative stress in animals fed Cont− than in those fed Cont+.

Prevention of the oxidation of LDL is an important effect of olive polyphenols in protection against cardiovascular diseases in humans. In the present study, the plasmatic level of oxLDL was not affected by any of the supplements used compared to both Cont− and Cont+. This does not comply with the results reported by Paiva-Martins et al. [3], who observed a trend of dietary olive leaves to lower oxLDL in pigs, although the supplementation level of polyphenols was much higher than in the present trial. The authors related the outcome to the increased level of vitamin E in olive leaves, while in our study, dietary supplementation with vitamin E did not have an effect on oxLDL. Our results are also different than those reported in humans. For instance, EFSA [2] substantiated the health claim that 5 mg of hydroxytyrosol and its derivatives per day protect LDL from oxidation, which is comparable to the concentration of polyphenols in group OLE-1. However, we observed a numerical difference between the OLE-supplemented and non-supplemented groups. The lack of statistical difference might have been attributed to the variability of oxLDL within the groups. In its trials, EFSA [2,16] supported the health claim because the difference in plasma oxLDL between the pre- and the post-intervention with polyphenol-rich olive oil was only 6.5%, while in our trial, OLE lowered the oxLDL level for approximately 20%, regardless of the concentration. The latter suggests that even the lowest concentration of OLE was sufficient to prevent LDL from oxidation. Nevertheless, a larger scale trial is suggested in order to confirm this hypothesis.

Dietary linseed oil (Cont+ vs Cont−) increased plasma MDA levels (*p* < 0.05) and showed a tendency to increase urinary MDA excretion levels (*p* = 0.115). This is in accordance with the previous findings of Frankič [6,17] and Frankič and Salobir [11], who discovered that dietary linseed oil increased plasma and urinary MDA levels in pigs in comparison to a diet without linseed oil. In the present study, the dietary inclusion of OLE did not affect plasma and urinary MDA levels, similarly to the results reported by Andreadou et al. [18] in rabbits. These authors observed that the dietary inclusion of oleuropein did not affect baseline MDA levels, but lowered plasma MDA levels when the oxidative stress was induced by ischemia and reperfusion. It is likely that ischemia and reperfusion had a stronger influence on the oxidative stress than the PUFA-rich diet in the present study, and therefore the effect of olive polyphenols could be different. Similarly, Al-Azzawie and Alhamdani [19] reported that dietary oleuropein in alloxan-diabetic rabbits reduced plasma MDA levels to the level of non-diabetic animals. As in previous studies by Frankič et al. [6,17], supranutritional vitamin E did not affect plasma and urinary MDA levels in piglets fed a PUFA-rich diet. This was an interesting result, since vitamin E is known as one of the best lipid-soluble antioxidants. 

Buckley et al. [20] reported that plasmatic tocopherols firmly follow the concentration of dietary tocopherols. Similarly, in our study, there was a higher concentration of γ-tocopherol in the plasma of animals fed diets supplemented with linseed oil (rich in γ-tocopherol) than in those fed Cont−. Moreover, dietary α-tocopheryl acetate (group Vit-E) significantly raised the plasmatic concentration of α-tocopherol and lowered that of γ-tocopherol within the linseed oil supplemented groups (Cont+, Vit-E, OLE-1, OLE-2, OLE-3). This might be related to the preferential transfer of α-tocopherol to lipoproteins and of γ-tocopherol to the bile by the liver, as observed in humans [21]. Furthermore, OLE did not have an effect on plasmatic α- and γ-tocopherols, unlike in the study by Paiva-Martins et al. [3], who reported that the dietary supplementation of olive leaves increased the content of α-tocopherol in muscle in pigs. This could be related to the higher level of α-tocopherol in the leaves than in OLE, and/or to the higher level of the supplementation compared to our study. 

In the present study, we included antioxidants with different polarities expecting different in vivo ACW and ACL. However, although some trials reported a positive change in plasmatic ACW and ACL in animals with dietary supplementation of antioxidants [22,23], we could not detect any differences in serum ACW and ACL between the groups. It is possible that the endogenous antioxidants were counteracting the presence of the exogenous antioxidants in order to retain the equilibrium of the total antioxidative capacity [24]. The unresponsiveness could also be attributed to a low absorption, strong homeostasis, fast metabolism and/or rapid excretion of polyphenols [12].

Glutathione peroxidase and SOD are among the most important enzymes involved in the antioxidant defense system [25]. In our study, neither dietary linseed oil nor OLE or vitamin E affected their activities, which is contrary to the positive effects of olive polyphenols on the expression of GPx [25,26] and SOD [27] reported by some in vitro studies. This could show a discrepancy between the in vivo and the in vitro trials, since the concentrations of the active compounds and their activities often differ between the two. Some authors reported an increase on the activities of GPx and SOD caused by dietary olive polyphenols in rats [28] and in humans [29,30]. However, the animals in our trial might have reacted differently, since we included high amounts of PUFA that increased the oxidative load in comparison to the animals and humans in other trials.

The oxidative products of n-3 PUFA can form adducts with DNA, suggesting their involvement in the pathogenesis of different diseases [31]. In our study, the inclusion of linseed oil (Cont+) caused higher DNA damage measured as DNA tail % in comparison to Cont−. This complies with previous results in a study on piglets, in which an increase in DNA damage was observed with the dietary inclusion of linseed oil [6,11,17]. In our trial, diet OLE-1 showed some protective effects on DNA measured as tail DNA % in comparison to Cont+ (*p* = 0.072), although the effect was not present in higher concentrations of OLE (OLE-2 and OLE-3). This partly agrees with some in vitro [32]) and in vivo [30] studies showing the protective effects of olive polyphenols against DNA damage. Interestingly, vitamin E presented prooxidative effects, as we detected higher OTM in the blood of animals fed Vit-E than in those fed Cont+, which does not comply with the previous findings of Frankič and Salobir [11]. These authors reported that dietary vitamin E completely mitigated the negative effects of dietary linseed oil on DNA damage in piglets, although the level of supplementation with vitamin E was lower than in our study.

The urinary level of 8-OHdG is a common marker of DNA damage or its repair [13]. In the present study, piglets fed Vit-E and OLE-1 had a similar excretion of 8-OHdG as piglets fed Cont−, which may suggest that the extent of DNA repair and/or damage was not altered in these groups. Contrary to our results, studies concerning DNA damage reported a reduction of 8-OHdG with dietary supplementation of olive polyphenols in humans [30]. The latter suggests that olive polyphenols might not be efficient in preventing DNA damage caused by PUFA-induced oxidative stress, or that the concentration of polyphenols might not have been sufficient to protect against DNA damage.

Lipids are primarily metabolized by the liver, and therefore its enzymes are an important marker of oxidative stress caused by n-3 PUFA. In our study, the inclusion of linseed oil (Cont+ vs. Cont−) increased the GGT activity in serum, suggesting an effect of the high intake of PUFA on liver function. Animals fed OLE-1 and Vit-E had lower GGT activity (*p* < 0.05 and *p* = 0.078, respectively) than those fed Cont−. This could indicate protective effects of OLE-1 and Vit-E, as demonstrated for other dietary antioxidants [33]. However, the differences between the latter and Cont+ were not significant (*p* > 0.05). In the present study, the activities of AST and ALT were not influenced by any of the supplements used, which is in agreement with the study by Bock et al. [34]. These authors reported no effects of olive polyphenols in plasmatic AST, ALT and GGT in humans. The results suggested that olive polyphenols do not have an apparent effect on the liver function, even in the case of high n-3 PUFA intake.

Antioxidants commonly have a concentration-dependent effect on the prevention of oxidative damage. Thus, we hypothesized that this could also be the case for OLE. Even though there are some reports on the concentration-dependent effects of olive polyphenols in pigs [3], turkeys [35], humans [16] and human erythrocytes [36], we did not obtain similar results. The lack of a clear dose-dependent antioxidative effect could be attributed to numerous nutritional and environmental factors. For instance, the oxidative stress caused by the high dietary intake of PUFA might have been overestimated, and the additional dietary antioxidant supplementation might have been unable to have an apparent effect even when included in high concentrations. Oxidative stress is also influenced by the duration of the stress: long stress periods could influence oxidative-induced damages in a substantially different way than short-term stress. Data on this are scarce, although some trials reported that a high intake of PUFA caused a rapid increase in the markers of oxidative stress in pigs [37]. Another important factor involved in the response to oxidative stress is the age of the animals. In our study, the animals were very young and could have had antioxidative defense systems with higher adaptability, antioxidative capacity and lower basal oxidative stress-induced damages, as is the case in humans [24,38]. Furthermore, the basal supplementation level of OLE was that recommended to prevent the oxidation of LDL in humans [2]. Hence, the response of humans and pigs could be different due to differences in metabolism, nutrition, age and genetic predispositions [39]. Previous studies in animals used higher amounts of olive polyphenols than in our study. 

## 5. Conclusions

In conclusion, the lowest level of the dietary OLE affected n-3 PUFA-induced oxidative stress measured as 8-OHdG excretion, DNA tail % and liver GGT in piglets. The supranutritional level of vitamin E did not show clear effects on lipid peroxidation and DNA damage, even though positive effects on the excretion of 8-OHdG and on the hepatic GGT were observed. Despite some positive effects of OLE on oxidative stress reduction, the results did not show clear concentration dependence and should be further tested to obtain optimal dietary concentrations. Since growth and metabolic rate are age dependent, the effects of OLE might be stronger in older animals, a longer supplementation period, and/or ad libitum feeding.

## Figures and Tables

**Table 1:** Composition, analyses and fatty acid content of the experimental diets.

Component	Cont−	Cont+, Vit-E, OLE-1, OLE-2, OLE-3
Composition (g/kg)		
Wheat starch	379.37	219.82
Soybean meal	220.75	247.87
Skimmed milk powder	125.69	141.15
Full-fat milk powder	61.82	69.42
Maize	112.56	126.38
Wheat bran	47.05	52.83
Rapeseed oil	18.99	21.32
Linseed oil	-	83.30
Sodium chloride	2.43	2.73
Limestone	9.72	10.92
Monocalcium phosphate	8.67	9.73
L-lysine	4.02	4.51
DL-methionine	2.35	2.63
Threonine	1.80	2.02
Premix ^1^	4.78	5.37
Chemical composition of diets (g/kg)		
Dry matter	903	917
Crude protein	184.4	205.3
Crude fat	37.28	125.21
Crude ash	46.96	52.03
NDF ^2^	67.91	72.85
ADF ^3^	23.49	23.96
Ca	8.83	9.67
P	5.85	6.44
Metabolisable energy (MJ/kg)	14.99	16.84
Fatty acids (g/kg) ^4^		
C16:0	6.09	12.09
C18:0	1.68	5.55
C18:1	13.84	34.38
C18:2 n-6	8.08	21.30
C18:3 n-3	1.55	39.59
n-6 PUFA ^5^	8.12	21.41
n-3 PUFA ^5^	1.55	39.62
n-6/n-3 PUFA ^5^	5.24	0.54
Sum of SFA ^6^	11.05	22.12
Sum of MUFA ^7^	14.63	35.71
Sum of PUFA ^5^	9.73	61.12

Cont− = negative control group; Cont+ = positive control group; Vit-E = DL-α-tocopheryl acetate-supplemented group; OLE-1 = OLE calculated following European Food Safety Authority (EFSA) recommendations; OLE-2 = OLE in 10× EFSA recommendations; OLE-3 = OLE in 25× EFSA recommendations. ^1^ 2000 IU of vitamin A/kg feed, 12.5 IU vitamin E/kg feed and 0.20 mg Se/kg feed. ^2^ Neutral detergent fiber. ^3^ Acid detergent fiber. ^4^ Only predominant fatty acids are shown. ^5^ Polyunsaturated fatty acid. ^6^ Saturated fatty acid. ^7^ Monounsaturated fatty acid.

**Table 2:** Daily intake of feed, energy and antioxidants calculated for a 10-kg piglet.

Daily Intake	Diets					
	Cont−	Cont+	Vit-E	OLE-1	OLE-2	OLE-3
Feed (g) ^1^	473.1	421.4	421.4	421.4	421.4	421.4
Hydroxytyrosol EQ (mg) ^2^	-	-	-	3.84	38.4	96.0
α-tocopherol (mg)	12.91	10.65	102.40	11.77	12.17	12.22
γ-tocopherol (mg)	4.28	17.13	17.96	17.96	18.50	17.72
Vitamin E (IU) ^3^	16.8	12.3	105.4	14.8	15.4	15.5
Energy from fat (%) ^4^	11.2	29.4	29.4	29.4	29.4	29.4
Energy from PUFA (%) ^4^	2.9	15.0	15.0	15.0	15.0	15.0
Energy from n-3 PUFA (%) ^4^	0.6	10.4	10.4	10.4	10.4	10.4

Nomenclature as in Table 1. ^1^ Calculated following the energy requirements of a 10-kg piglet (2.5× maintenance energy requirement) ^2^ Calculated from hydroxytyrosol equivalents (EQ) and its derivatives in OLE. ^3^ Calculated as 1 IU = 1 mg of DL-α-tocopheryl acetate; 0.67 mg of D-α-tocopherol, and 10 mg of γ-tocopherol. ^4^ Calculated values as % of total energy.

**Table 3:** Growth performance of piglets fed the experimental diets (*n* = 8/group).

Parameter	Group						*p*-Value
	Cont−	Cont+	Vit-E	OLE-1	OLE-2	OLE-3	
Initial body weight (kg)	10.6 ± 0.25	10.6 ± 0.25	10.6 ± 0.23	10.6 ± 0.21	10.7 ± 0.19	10.6 ± 0.22	0.996
Final body weight (kg)	17.8 ± 0.28	17.9 ± 0.33	18.2 ± 0.30	17.9 ± 0.21	17.7 ± 0.26	17.9 ± 0.31	0.823
Average daily gain (g)	346 ± 4.9	348 ± 8.0	360 ± 6.1	344 ± 9.9	330 ± 9.1	349 ± 5.6	0.169
Average daily feed intake (g)	535 ± 6.0 ^b^	477 ± 5.9 ^a^	481 ± 4.9 ^a^	479 ± 5.6 ^a^	477 ± 4.2 ^a^	479 ± 5.3 ^a^	<0.001
Conversion ratio ^1^	1.55 ± 0.023 ^b^	1.37 ± 0.030 ^a^	1.34 ± 0.021 ^a^	1.40 ± 0.048 ^ab^	1.45 ± 0.040 ^ab^	1.38 ± 0.014 ^a^	<0.001

Nomenclature as in Table 1. ^1^ Calculated as total feed intake/total weight gain. Values within the same row with different superscripts differ significantly (*p* < 0.05).

**Table 4:** Plasma and urinary markers of oxidative stress in piglets fed the experimental diets (*n* = 8/group).

Parameter	Group						*p*-Value
	Cont−	Cont+	Vit-E	OLE-1	OLE-2	OLE-3	
Urinary F_2_-isoprostanes (ng/24 h) ^1^	1961 ± 165.9 ^b^	1496 ±130.5 ^ab^	1270 ± 144.5 ^a^	1614 ± 204.2 ^ab^	1456 ± 232.9 ^ab^	2086 ± 356.8 ^ab^	0.0483
Serum oxLDL (nmol/mL) ^2^	171 ± 17.0	169 ± 10.1	167 ± 13.5	139 ± 25.5	132 ± 23.0	136 ± 13.4	0.2734
Plasma MDA (nmol/mL) ^3^	0.596 ± 0.045 ^a^	0.863 ± 0.042 ^bd^	0.670 ± 0.058 ^ab^	0.873 ± 0.032 ^cd^	0.864 ± 0.064 ^bc^	0.859 ± 0.057 ^bc^	0.0001
Urinary MDA (µmol/24 h) ^3^	5.57 ± 0.454	7.62 ± 0.622	5.92 ± 0.624	7.83 ± 0.710	5.80 ± 0.734	7.62 ± 0.553	0.0169

Nomenclature as in Table 1. ^1^ Urinary iPF2α-III. ^2^ Oxidized low-density lipoprotein. ^3^ Malondialdehyde. Values within the same row with different superscripts differ significantly (*p* < 0.05).

**Table 5:** Levels of plasma α- and γ-tocopherol, serum antioxidant capacities of water- and lipid-soluble antioxidants and antioxidant enzyme activities in piglets fed the experimental diets (*n* = 8/group).

Parameter	Group						*p*-Value
	Cont−	Cont+	Vit-E	OLE-1	OLE-2	OLE-3	
Plasma α-tocopherol (µg/mL)	1.74 ± 0.143 ^a^	1.54 ± 0.118 ^a^	4.52 ± 0.221 ^b^	1.60 ± 0.169 ^a^	1.58 ± 0.191 ^a^	1.39 ± 0.071 ^a^	<0.001
Plasma γ-tocopherol (µg/mL)	0.084 ± 0.011 ^a^	0.546 ± 0.046 ^c^	0.209 ± 0.019 ^b^	0.460 ± 0.064 ^c^	0.413 ± 0.046 ^c^	0.346 ± 0.052 ^bc^	<0.001
Serum ACW (nmol/mL) ^1^	65.7 ± 3.35	67.8 ± 2.92	73.8 ± 2.42	78.7 ± 5.00	79.8 ± 3.70	71.1 ± 3.31	0.0499
Serum ACL (nmol/mL) ^2^	112 ± 3.78	123 ± 5.57	115 ± 4.65	123 ± 4.22	123 ± 7.20	125 ± 5.20	0.2143
Whole blood GPx (U/g HGB) ^3^	261 ± 12.5	296 ± 26.1	289 ± 12.9	241 ± 12.2	249 ± 6.0	273 ± 14.8	0.0404
Whole blood SOD (U/g HGB) ^4^	1839 ± 65.3	1781 ± 72.0	1687 ± 106.5	1743 ± 68.0	1793 ± 110.3	1858 ± 72.9	0.7215

Nomenclature as in Table 1. ^1^ Antioxidant capacity of water-soluble antioxidants. ^2^ Antioxidant capacity of lipid-soluble antioxidants. ^3^ Glutathione peroxidase. ^4^ Superoxide dismutase. Values within the same row with different superscripts differ significantly (*p* < 0.05).

**Table 6:** DNA damage markers and liver enzymes activities in piglets fed the experimental (*n* = 8/group).

Parameter	Group						*p*-Value
	Cont−	Cont+	Vit-E	OLE-1	OLE-2	OLE-3	
Tail DNA (%)	7.91 ± 0.257 ^ad^	9.07 ± 0.305 ^bc^	9.75 ± 0.324 ^b^	7.92 ± 0.298 ^cd^	8.88 ± 0.307 ^abc^	8.38 ± 0.300 ^ac^	<0.0001
OTM^1^	0.730 ± 0.032 ^a^	0.830 ± 0.031 ^a^	0.986 ± 0.032 ^b^	0.729 ± 0.030 ^a^	0.820 ± 0.032 ^a^	0.842 ± 0.030 ^a^	<0.0001
Urinary 8-OHdG (µg/24 h) ^2^	418 ± 41.7 ^bc^	253 ± 26.9 ^ad^	389 ± 31.2 ^c^	345 ± 28.3 ^bcd^	237 ± 31.9 ^ad^	236 ± 18.9 ^a^	0.0001
Serum AST (U/L) ^3^	47.6 ± 3.50	64.5 ± 6.28	47.6 ± 3.73	58.5 ± 7.31	58.3 ± 6.86	47.8 ± 4.74	0.1370
Serum ALT (U/L) ^4^	85.0 ± 9.09	93.9 ± 7.95	92.8 ± 8.72	98.1 ± 7.47	95.5 ± 7.35	82.6 ± 6.36	0.6070
Serum GGT (U/L) ^5^	37.0 ± 2.01 ^a^	51.9 ± 4.09 ^b^	35.6 ± 4.13 ^ab^	38.1 ± 1.65 ^a^	44.5 ± 4.66 ^ab^	46.0 ± 2.42 ^ab^	0.0051

Nomenclature as in Table 1. ^1^ Olive tail moment. ^2^ 8-hydroxy-2’-deoxyguanosine. ^3^ Aspartat aminotransferase. ^4^ Alanine aminotransferase. ^5^ Gamma-glutamyl transferase. Values within the same row with different superscripts differ significantly (*p* < 0.05).
